# Characterizing TIA and stroke symptomatology in a population-based study: implications for and diagnostic value of FAST-based public education

**DOI:** 10.1186/s12889-024-20960-5

**Published:** 2024-12-18

**Authors:** Jacqueline J. Claus, Bernhard B. P. Berghout, Camiel V. J. Box, Silvan Licher, Bob Roozenbeek, M. Kamran Ikram, Frank J. Wolters

**Affiliations:** 1 Department of Epidemiology, Erasmus MC, University Medical Centre Rotterdam, Wytemaweg 80, Rotterdam, CA 3015 the Netherlands; 2 Department of Radiology & Nuclear Medicine, Erasmus MC, University Medical Centre Rotterdam, Wytemaweg 80, Rotterdam, CA 3015 the Netherlands; 3 Department of Neurology, Erasmus MC, University Medical Centre Rotterdam, Wytemaweg 80, Rotterdam, CA 3015 the Netherlands; 4 Department of General Practice, Erasmus MC, University Medical Centre Rotterdam, Wytemaweg 80, Rotterdam, CA 3015 the Netherlands

**Keywords:** Stroke, TIA, FAST, Stroke recognition, Stroke prevention

## Abstract

**Background:**

Urgent medical treatment is crucial after stroke and transient ischemic attack (TIA), but hindered by extensive prehospital delays. Public education campaigns based on FAST (Face-Arm-Speech-Time) have improved response after major stroke, but not minor stroke and TIA. We aimed to provide strategies to improve public education on a national level, by characterizing TIA and stroke symptoms in a population-based cohort, and extrapolating findings to the general Dutch population.

**Methods:**

We included all patients with first-ever stroke or TIA from 2002–2016 in the population-based Rotterdam Study (*N* = 17,931). We determined the prevalence of focal neurological symptoms and their combinations by event severity (i.e., TIA, minor stroke [National Institutes of Health Stroke Scale (NIHSS) 0–3], and major stroke [NIHSS > 3]). We assessed sensitivity of the FAST test for TIA and stroke, and estimated specificity using survey data on the incidence of focal neurological symptoms of non-vascular origin from the same source population. Finally, we determined the diagnostic value of adding visual symptoms and vertigo to the FAST test.

**Results:**

Of all 900 patients (mean age: 77.6 years, 57.2% women), 409 (45.4%) had a TIA, 254 (28.2%) had minor stroke, and 237 (26.3%) had major stroke. At least one FAST symptom was present in 233/237 (98.3%) of patients with major stroke, compared to 186/254 (73.2%) patients with minor stroke, and 250/402 (62.2%) with TIA. Minor strokes and TIA not captured by the FAST test most commonly involved visual symptoms (52.7%), dizziness/vertigo (19.5%), disturbed coordination (19.1%), and sensory disturbance (18.2%). Sensitivity of FAST for TIA/minor stroke increased from 66.4 to 80.8% with the addition of visual symptoms, and to 86.1% with further incorporation of dizziness/vertigo, albeit with a > 40% increase in the number of false positive events. Nearly all patients with major stroke (97.5%) experienced a combination of multiple symptoms, whereas 58.9% of patients with TIA and 26.4% of those with minor stroke reported only a single symptom.

**Conclusions:**

In contrast to major stroke, sensitivity of the FAST test is limited to around 65% for TIA and minor stroke in a population-based setting. Sensitivity increases by incorporating visual symptoms and vertigo, but this comes with a large number of false positives. Findings of this study may favor a focus on the importance of isolated or transient symptoms, rather than additional symptoms, in future stroke public education campaigns.

**Supplementary Information:**

The online version contains supplementary material available at 10.1186/s12889-024-20960-5.

## Introduction

Risk of early recurrent stroke is high after transient ischemic attack (TIA) and minor stroke, with up to 10% of patients having recurrent stroke within a week when left untreated [1]. Urgent medical treatment reduces risk of early recurrent stroke by 80% [2, 3], but is often hindered by prehospital delays [4, 5]. Two-thirds of patients with TIA and minor stroke do not perceive their symptoms as such, and one-third delay seeking medical attention beyond 24 h.[6] Moreover, approximately half of all recurring strokes are preceded by a TIA for which no medical attention was sought [7]. In ischemic stroke, the prompt initiation of reperfusion therapy corresponds to improvement in clinical outcomes for both minor and major stroke [8, 9]. Public education campaigns have aimed to improve symptom recognition and timely medical attention after stroke. The Face-Arm-Speech-Time (FAST) test has formed the basis of public education in the United Kingdom, Ireland, United States, Australia, and New Zealand, with variants in several non–English-speaking countries [7, 10–13]. While such FAST-based campaigns have been associated with improved response to major stroke, patient response to TIA and minor stroke has remained unchanged [7, 14].

The lack of effectiveness of FAST-based campaigns to improve response after TIA and minor stroke may be attributable, at least in part, to a lower sensitivity of the FAST test for minor events. Indeed, a population-based study in the United Kingdom found that around one-third of TIA and minor stroke events were not captured by the FAST acronym [7]. Effectiveness of public education might improve by focus on additional stroke symptoms, but it remains uncertain which combination of symptoms would be most informative to alternative education strategies. One study among patients with mostly major stroke in a comprehensive stroke center, observed that extension of the FAST-acronym with visual symptoms and balance disturbance increased sensitivity from 86 to 96% [15]. However, no published studies have assessed the value of expansions to FAST among patients with minor stroke and TIA in a population-based setting. Other reasons for the limited effectiveness of public education on TIA and minor stroke presentations may include the focus of education efforts on disabling and persistent complaints. Better characterization of transient and non-disabling neurological symptoms on a population level could be helpful to tailor public education strategies also to these minor events, which comprise two-thirds of all cerebrovascular events in the general population [7].

We therefore aimed to determine the sensitivity of the FAST-test in the general population, stratified by TIA and stroke severity, and characterized symptomatology. To inform future public education campaigns, we extrapolated findings from the population-based Rotterdam Study to the general Dutch population, aiming to assess the diagnostic value of public education on a national scale.

## Methods

### Study population

This study was embedded within the Rotterdam Study, an ongoing population-based study of determinants and occurrence of disease in persons aged 40 years and older. The study comprises 17,931 individuals living in the Ommoord suburb of Rotterdam, the Netherlands [16]. The design of the Rotterdam Study has been described in detail previously. In brief, participants are invited for interview and extensive in-person examination at a dedicated research center about once every 3–6 years. The current study includes all participants with first-ever TIA and stroke (with the exception of subarachnoid hemorrhage) between 1st April 2002 and 31st December 2016. Participants with prevalent dementia (*n* = 144) or decreased consciousness (*n* = 91) were excluded (Supplemental Fig. 1). The report is following STROBE (Strengthening the Reporting of Observational Studies in Epidemiology) guidelines for cohort studies.

### Ethics approval

The Rotterdam Study has been approved by the Medical Ethics Committee of the Erasmus MC and by the Ministry of Health, Welfare and Sport of the Netherlands, implementing the Population Screening Act: Rotterdam Study. All participants provided written informed consent to participate in the study and to obtain information from their treating physicians.

### Availability of data

Data can be obtained upon request. Requests should be directed towards the management team of the Rotterdam Study (secretariat.epi@erasmusmc.nl), which has a protocol for approving data requests. Because of restrictions based on privacy regulations and informed consent of the participants, data cannot be made freely available in a public repository. FJW had full access to the data in the study and takes responsibility for data integrity and accuracy of data analysis.

### Ascertainment of stroke and TIA

Stroke was defined according to the World Health Organization criteria as a syndrome of rapidly developing clinical signs of focal (or global) disturbance of cerebral function, with symptoms lasting 24 h or longer or leading to death, with no apparent cause other than of vascular origin [17]. We defined TIA as the presence of focal neurological symptoms, which lasted no longer than 24 h and were attributable to dysfunction of one arterial territory of the brain [18]. In addition to invited examinations, participants were continuously monitored for the occurrence of stroke and TIA through linkage of the study database with files from general practitioners and nursing home physicians, which included discharge letters from any hospital admission or outpatient visit. Potential TIA and stroke cases were reviewed by research physicians, and an experienced vascular neurologist adjudicated the final diagnosis, as described in detail previously [19]. A research physician manually reviewed all medical charts and noted the presence of different neurological symptoms (i.e., facial palsy, arm and leg weakness, speech disturbance, visual symptoms, disturbed coordination, sensory disturbance, and dizziness/vertigo). We did not distinguish between proximal and distal arm or leg paresis. Any unmentioned symptoms were presumed absent, in accordance with a previously validated method for assessing stroke severity [20]. Stroke severity was evaluated by reviewing medical charts, applying this previously validated methodology that standardizes record-based NIHSS assessment using a predefined scoring rule. This approach ensures consistent interpretation of documented symptoms to assign NIHSS scores and differentiate between minor and major strokes [20]. The only exception to this rule were very severe hemispheric strokes, for which commonly present symptoms that often remain unmentioned in medical charts were marked present (e.g., neglect and hemianopia) [20]. Major stroke was defined as an NIHSS > 3.

### Questionnaire study on stroke symptoms

Between April and July 2020, a series of questionnaires was sent out to all community-dwelling participants of the Rotterdam Study. The questionnaires were sent both digitally and on paper, leading to an average response rate of 73.5%. For the current analysis we used data from the sixth survey, which included questions on the occurrence of focal neurological symptoms during the past 2 months. At total of 4,705 participants filled in the questionnaire, of whom 3,854 completed the questions on the occurrence of neurological symptoms and were aged 50 years or older. Participants were specifically enquired about sudden limb weakness, facial droop, speech difficulties, vertigo, transient loss of vision, and a numb or tingling sensation anywhere in the body (Supplemental Table 1).

### Statistical analyses

We first compared patient characteristics between TIA, minor stroke (NIHSS ≤ 3), and major stroke (NIHSS > 3) from the Rotterdam Study cohort, focusing on age, sex, stroke subtype and TIA duration of symptoms.

We calculated prevalence of focal neurological symptoms in patients with TIA, minor stroke, and major stroke. Events were denoted as FAST-positive, if patients had facial droop, speech difficulty, and/or arm weakness. We calculated the number of FAST-symptoms for all events in the Rotterdam Study cohort, and determined the sensitivity of the FAST test for TIA as well as minor and major stroke.

Next, we mapped the occurrence of different combinations of symptoms, again stratified by event severity, using events from the Rotterdam Study cohort. We subsequently determined the change in sensitivity of the FAST test, if it would additionally incorporate visual symptoms (‘eyes’; E-FAST) and visual symptoms plus dizziness/vertigo (‘balance and eyes’; BE-FAST).

We used the reported incidence of focal neurological symptoms over a 2-month time period in the Rotterdam Study questionnaire to estimate the number of persons aged 50 years and older in the study expected to experience any of these symptoms. We extrapolated this incidence rate to the general Dutch population, using population data from Statistics Netherlands, by multiplying the incidence rate with the total number of persons in the Dutch population aged 50 years and older [21].

Similarly, using the incidence rate of stroke and TIA in all participants from the Rotterdam Study aged 50 years and older between 2010–2020 (11.95 per 1000 person years), we estimated the expected number of cerebrovascular events in the general population during 2 months. Subsequently, we used these numbers to estimate the diagnostic value (i.e., specificity, positive and negative predictive value) of the FAST-test, as well as E-FAST and BE-FAST expansions.

All analyses were performed in R (version 4.2.1), and plots were created using the “upset” package.

## Results

Of 900 patients with first-ever TIA or stroke, 409 (45.4%) had TIA, 254 (28.2%) had minor stroke, and 237 had (26.3%) major stroke. Mean age of patients was 77.6 (± 9.4) years and 57.2% were female (Table 1). Of 311 TIAs for which duration of symptoms was reported, two-thirds (211/311; 67.8%) lasted less than 1 h.

**Table 1:** Demographic and clinical characteristics for TIA, minor stroke (NIHSS ≤ 3), major stroke (NIHSS > 3)

	TIA	Minor stroke	Major stroke
	*N* = 409	*N* = 254	*N* = 237
Age, mean (**± **SD)	76.7 (9.9)	77.4 (8.8)	79.5 (8.9)
Sex, female	245 (59.9)	145 (57.1)	125 (52.7)
Stroke subtype			
Ischemic	409 (100.0)	208 (84.2)	170 (75.6)
Haemorrhagic	n/a	11 (4.5)	28 (12.4)
Undetermined	n/a	28 (11.3)	27 (12.0)
Symptom duration			
0–59 s	34 (10.9)	n/a	n/a
1–59 min	177 (56.9)	n/a	n/a
1–24 h	100 (32.2)	n/a	n/a
> 24 h	n/a	254 (100.0)	237 (100.0)

Numbers are *N* (%) unless specified otherwise

Data were missing for level of education (1.6%), stroke subtype (3.9%) and TIA duration of symptoms (24.0%)

*NIHSS* National Institutes of Health Stroke Scale, *TIA* Transient ischemic attack, *SD* Standard deviation, and *n/a* not applicable

### Sensitivity of the FAST test

**Fig. 1:**
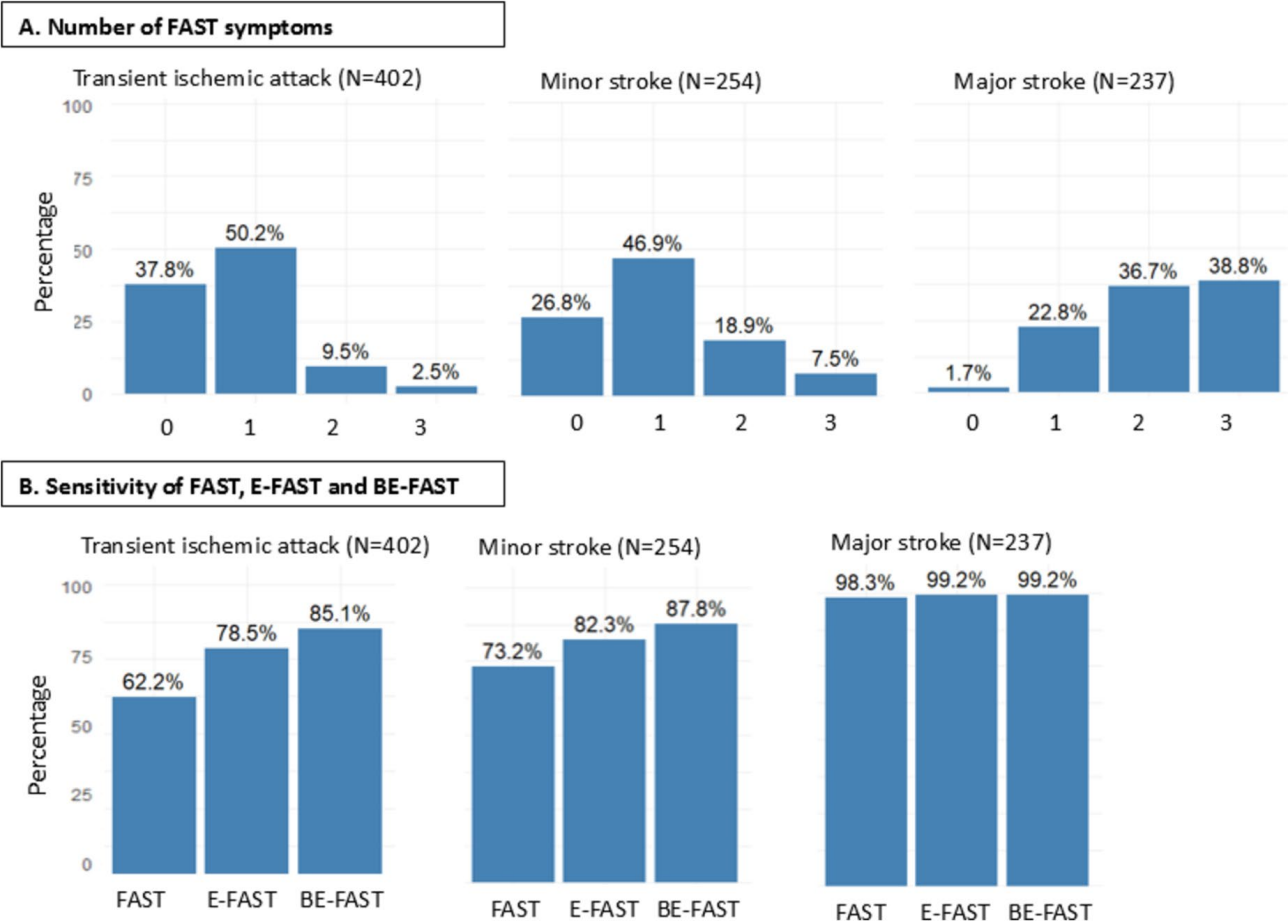
Prevalence of FAST symptoms according to TIA and stroke severity. Legend: Percentages reflect the percentage of patients from the Rotterdam Study, with (**A**) zero, one, two or three FAST symptoms, (**B**) at least one FAST symptom and expansions of the FAST-test by additionally including visual field defects (Eyes) and dizziness/vertigo (Balance). FAST indicates Face-Arm-Speech-Time; E-FAST Eyes-Face-Arm-Speech-Time; BE-FAST Balance-Eyes-Face-Arm-Speech-Time

Virtually all patients with major stroke had ≥ 1 FAST symptom (233/237, 98.3%), whereas ≥ 1 FAST-symptom was present in 186/254 (73.2%) minor strokes, and 250/402 (62.2%) TIAs (Fig. 1). All FAST symptoms were less common with minor stroke and TIA than with major stroke. For major stroke, arm weakness was present in 89% of patients, followed by speech disturbance in 71%, and facial palsy in 53% of events (Table 2). In contrast, speech disturbance was the most common FAST-symptom in TIA and minor stroke, present in 99/254 (39.0%) of minor strokes and 176/409 (43.0%) of TIAs. Arm weakness was present in 126/254 (49.6%) minor stroke and 96/409 (23.5%) TIA, and facial palsy in 47/254 (18.5%) of minor stroke and 36/409 (8.8%) of TIA. Sensitivity of the FAST-test was similar between men and women, with at least 1 FAST symptom present in 262/385 (68.1%) of women with TIA or minor stroke, and in 174/271 (64.2%) of men.

**Table 2:** Occurrence of neurological symptoms for all first TIA and strokes in the Rotterdam study

	TIA	Minor stroke	Major stroke	Respondents survey^a^
	*N* = 409	*N* = 254	*N* = 237	*N* = 3,854
Weakness	151 (36.9)	154 (60.6)	228 (96.2)	n/r
Facial palsy	36 (8.8)	47 (18.5)	126 (53.2)	39 (1.0)^b^
Arm weakness	96 (23.5)	126 (49.6)	211 (89.0)	303 (7.8)^b^
Leg weakness	69 (16.9)	89 (35.0)	183 (77.2)	303 (7.8)^b^
Language or speech deficit	176 (43.0)	99 (39.0)	167 (70.5)	39 (1.0)^b^
Sensory disturbance	58 (14.2)	68 (26.8)	80 (33.8)	243 (6.3)
Visual symptoms	100 (24.4)	46 (18.1)	62 (26.2)	181 (4.7)
Visual field defect	78 (19.1)	32 (12.6)	60 (25.3)	n/r
Diplopia	17 (4.2)	14 (5.5)	3 (1.3)	n/r
Positive visual phenomena	13 (3.2)	10 (3.9)	1 (0.4)	n/r
Dizziness	43 (10.5)	38 (15.0)	25 (10.5)	n/r
Vertigo	24 (5.9)	22 (8.7)	6 (2.5)	463 (12.0)
Non-rotatory dizziness	19 (4.6)	19 (7.5)	19 (8.0)	n/r
Disturbed coordination	37 (9.0)	33 (13.0)	25 (10.5)	n/r

Expressed as patients, N (%). TIA indicates transient ischemic attack

*TIA* transient ischemic attack, and *n/r *not recorded

^a^Respondents to the population-based survey of stroke symptoms, survey is described in Supplemental Table 1

^b^Survey inquired: “trouble speaking or drooping mouth” and “loss of strength in arm or leg”

### Non-FAST symptoms in stroke and TIA

Apart from FAST, the most common symptoms were leg weakness (24.1% TIA/minor stroke, 77.2% major stroke), visual symptoms (22.3% TIA/minor stroke, 26.2% major stroke), sensory disturbance (18.9% TIA/minor stroke, 33.8% major stroke), and dizziness/vertigo (12.3% TIA/minor stroke, 10.5% major stroke). Of all 208 patients with visual symptoms, 170 (82%) had visual field defects, with another 34 (17%) reporting diplopia. Of all 106 patients with dizziness, half were most consistent with vertigo and half with non-rotatory dizziness. These patterns were similar for TIA and minor stroke.

**Fig. 2:**
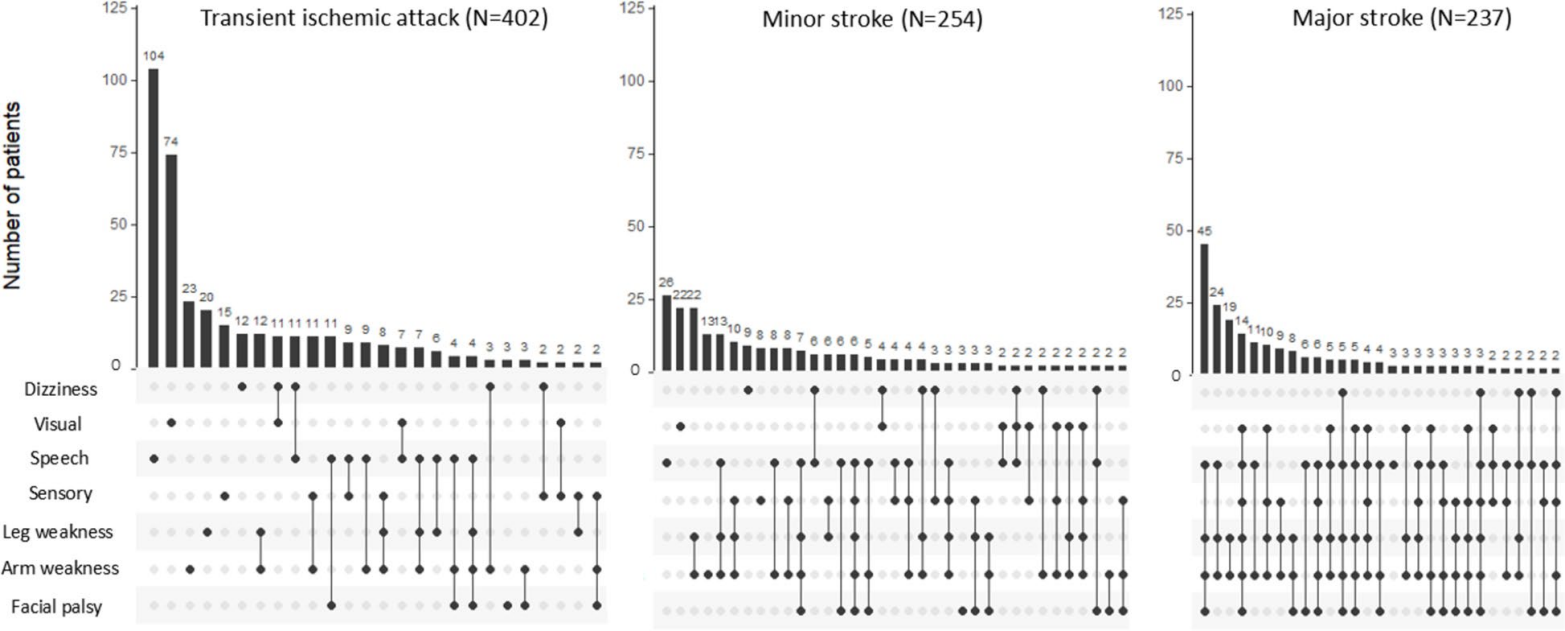
Concurrence of neurological symptoms by TIA and stroke severity. Legend: These intersection diagrams show the patterns of co-occurrence of different symptoms. Rows represent the types of symptom and columns represent their combinations. All symptoms that are part of a given combination are shown as black dots connected by a vertical black line. A single dot without a line implies the symptom occurred in isolation. The number of participants with a given combination of symptoms is shown as a vertical bar on top of the matrix. A minimum of two participants per combination is shown, single participant combinations are not shown

**Fig. 3:**
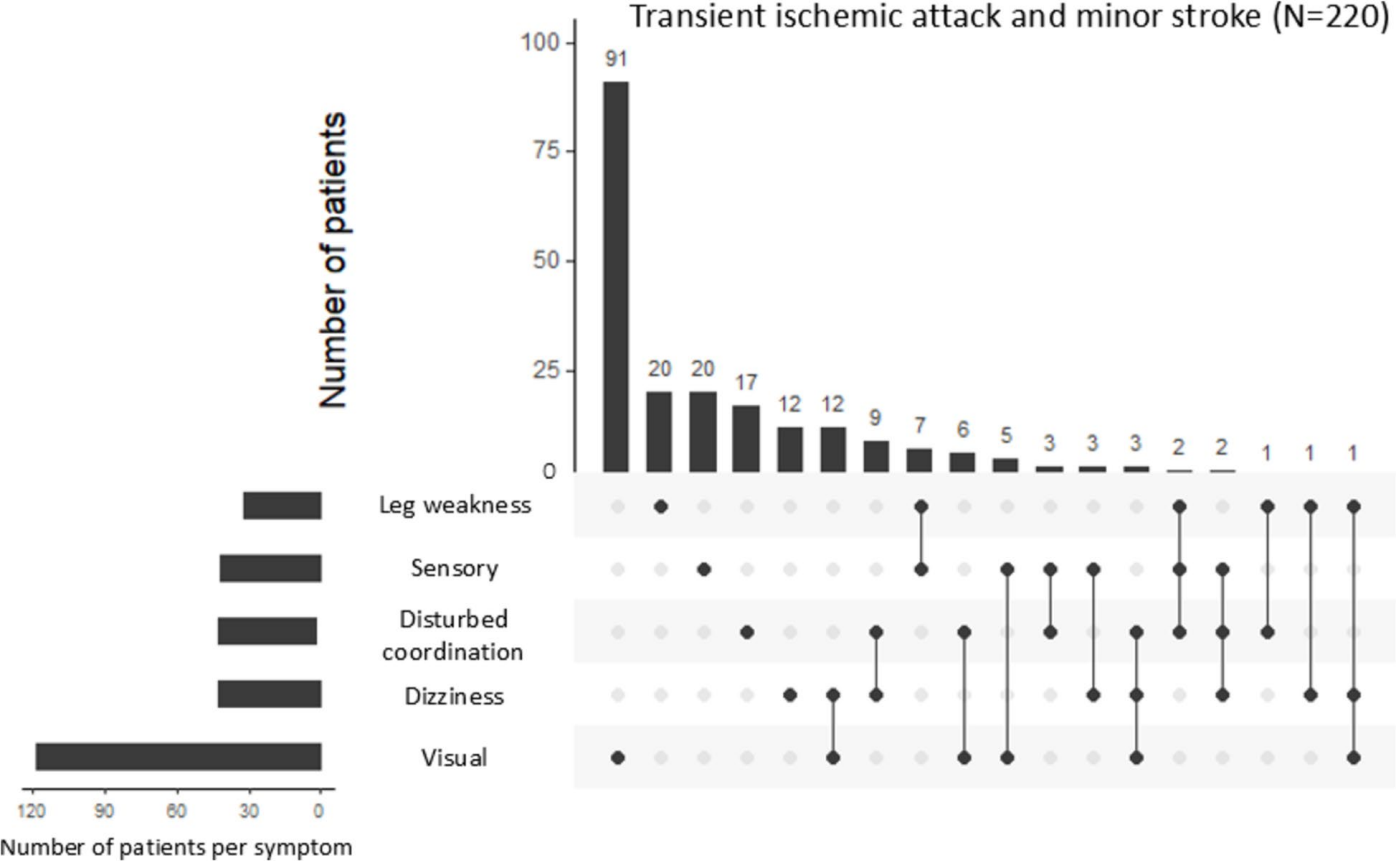
TIA and stroke symptoms in patients without any FAST symptom. Legend: This intersection diagram shows the patterns of co-occurrence of different symptoms. Rows represent the types of symptom and columns represent their combinations, sorted by frequency. All symptoms that are part of a given combination are shown as black dots connected by a vertical black line. A single dot without a line implies the symptom occurred in isolation. The number of participants with a given combination of symptoms is shown as a vertical bar on top of the matrix. All combinations of all participants are shown

Nearly all patients with major stroke experienced a combination of different symptoms (97.5%; Fig. 2). In contrast, a large proportion of patients with TIA, and to a lesser extent minor stroke, had isolated symptoms, most commonly consisting of speech disturbance (23.7% TIA and 10.2% minor stroke) or visual symptoms (15.9% TIA and 5.6% minor stroke). Of all 220 patients with TIA or minor stroke who did not experience any of the FAST symptoms, 116 (52.7%) had visual symptoms, 43 (19.5%) dizziness/vertigo and 42 (19.1%) disturbed coordination, with visual symptoms most often occurring in isolation (Fig. 3).

### Transient neurological symptoms in the general population

Of 3,854 participants who completed the questionnaire on neurological symptoms, 881 (22.9%) reported any neurological symptom during the past two months, i.e., sudden limb weakness, facial droop, speech difficulties, vertigo, transient loss of vision, or sensory symptoms. Of these, 326 (8.5%) participants reported any of the FAST symptoms (i.e., sudden limb weakness, speech difficulties or facial droop). Sudden transient loss of vision was reported by 181 individuals (4.7%), and vertigo or non-rotatory dizziness was reported by 463 (12.0%) participants (Table 2). Consequently, 456 respondents (11.8%) reported any FAST symptom or loss of vision, and 772 (20.0%) reported any FAST symptom, vertigo/dizziness, or transient loss of vision. Sensory symptoms were reported by 243 individuals (6.3%).

### Diagnostic value of an expanded FAST-test

Incorporation of visual symptoms into the FAST acronym increased sensitivity for TIA and minor stroke from 66.4% to 80.8% among 656 participants from the Rotterdam Study (Fig. 1). Additional incorporation of dizziness/vertigo further increased sensitivity for TIA and minor stroke to 86.1% (Fig. 1).

Table 3 shows the diagnostic value of the FAST-test, and potential expansions, by combining the observed sensitivity of the FAST-test within the Rotterdam Study, with the incidence of transient neurological symptoms and TIA/stroke from questionnaires, extrapolated to the Dutch population. Based on incidence rates in the Rotterdam Study, there are 87,525 strokes and TIAs occurring in the general population aged > 50 years in the Netherlands annually, implying 14,588 events during the 2-month questionnaire period. Of these, 10,926 would be FAST-positive (sensitivity = 74.9%) and 13,071 BE-FAST positive (sensitivity = 89.6%) during 2 months (Table 3).

**Table 3:** Estimated diagnostic value of the FAST test in the general Dutch population. Legend: Comparison of the FAST-acronym with additional incorporation of visual (eye) symptoms (E-FAST) and balance (dizziness/vertigo) and eye symptoms (BE-FAST)

	TIA and stroke	Stroke mimic^a^	Total^b^	Sensitivity	Specificity	PPV	NPV
FAST positive	10,926	611,637	622,563	74.9%	63.2%	1.8%	99.6%
FAST negative	3,662	1,051,032	1,054,694				
E-FAST positive	12,502	851,762	864,264	85.7%	48.8%	1.5%	99.7%
E-FAST negative	2,086	810,907	812,993				
BE-FAST positive	13,071	1,451,782	1,464,853	89.6%	12.7%	0.9%	99.3%
BE-FAST negative	1,517	210,887	212,404				
Total	14,588	1,662,669	1,677,257	-	-	-	-

622,563 (8.5%) are estimated to have experienced focal neurological symptoms included in FAST (limb weakness, speech difficulties or facial droop)

Incidence rates of TIA, stroke, and focal neurological symptoms of non-vascular origin from the population-based Rotterdam Study are projected on the population aged > 50 years in the Netherlands (according to census data by Statistics Netherlands)

*TIA* indicates transient ischemic attack, *PPV* positive predictive values, *NPV* negative predictive value, *FAST* Face-Arm-Speech-Time, *E-FAST* Eyes-Face-Arm-Speech-Time, *BE-FAST* Balance-Eyes-Face-Arm-Speech-Time

^a^Indicates symptoms of non-neurological origin

^b^Includes: facial droop, speech difficulties, limb weakness, vision loss and vertigo, and sensory symptoms, but no coordination problems as these were not inquired in the population-based survey of stroke symptoms

We extrapolated the incidence of transient neurological symptoms from the observed distribution in the questionnaire in the sample of 3,854 participants, to all 7,324,267 persons aged > 50 years living in the Netherlands. In the questionnaire 22.9% experienced any neurological symptom in the past 2 months, meaning 1,677,257 (22.9%) Dutch citizens are estimated to experience any focal neurological symptom per 2 months. Of those, 622,563 (8.5%) are estimated to experience FAST-positive focal neurological symptoms per 2 months (Table 3), 11.8% (864,264/7,324,267) are estimated to experience FAST-symptoms or loss of vision, and 20.0% (1,464,853/7,324,267) to experience FAST symptoms, visual field defects or vertigo.

The number of patients with stroke mimics was calculated by subtracting the expected number of TIA and stroke patients over a two-month period from the total number of individuals in the Netherlands who exhibited stroke-like symptoms during the same time frame. This led to a positive predictive value of the FAST-test for a cerebrovascular event of 1.8%, which declined to 1.5% for E-FAST, and 0.9% for BE-FAST (Table 3). Specificity for FAST was 63.2%, declining to 48.8% after incorporation of visual symptoms (E-FAST), and to 12.7% for BE-FAST. The number of false positive cases consequently increased 2.4-fold by expanding FAST to a BE-FAST acronym (Table 3).

## Discussion

In this population-based study of TIA and stroke patients, sensitivity of the FAST-test for detecting TIA and minor stroke was much lower than for major stroke, with one-third of TIA and minor stroke not captured by the FAST-test. FAST-negative events often involved visual symptoms or vertigo/dizziness, and incorporation of these symptoms in the FAST-acronym increased sensitivity from 66 to 86%, but at the cost of a large number of false positive cases and decreasing positive predictive value.

The lower sensitivity of the FAST-test for minor stroke and TIA, compared to major stroke, is in line with a prior population-based study from the United Kingdom [7]. This discrepancy between minor and major cerebrovascular events may be partly explained by posterior strokes, which are more frequently classified as minor due to less NIHSS points attributable to posterior symptoms [22, 23]. The FAST test has been shown to have lower sensitivity for detecting these posterior strokes [24]. Our findings explain why sensitivity of FAST is higher in cohorts of clinical stroke patients, which often include patients with more severe neurological deficits. In light of public education, this population-based perspective is of particular importance, as around 70% of all cerebrovascular events in the general population are minor stroke or TIA [7, 25]. When patients and bystanders comprehend the gravity of stroke symptoms, they are more likely to call for emergency services, leading to reduced prehospital delays [5, 26].

Among those with stroke mimics within the extrapolated data from our study 60% had no FAST-symptoms, aligning with the pooled estimated specificity of the FAST-test of 60% in nine clinical studies [27]. However, for the BE-FAST-test, specificity in clinical studies was higher compared to our specificity of 13%, ranging from 23 to 56% [28, 29]. Inclusion of patients presenting to emergency medical services in clinical studies, compared to our population-based survey, likely led to sampling of more severe cases of dizziness and vertigo, with higher probability of cerebrovascular disease [29, 30]. We estimated that incorporation of vision loss and vertigo in the FAST-test could more than double the number of presentations for suspected stroke. Given the high prevalence of vision loss and dizziness of non-cerebrovascular origin in the population, expansion of the FAST-test could imply a substantial increase in the burden on the healthcare system. This should be carefully weighed against potential benefits when designing new public education campaigns. We found that approximately one in five participants experienced at least one stroke symptom within the past two months. This is a relatively high figure, exceeding the lifetime prevalence of 18–30% reported in previous studies for stroke-like symptoms in individuals without a history of cerebrovascular events [31–33]. The discrepancy could be attributed to differences in assessment methods. Assessors conducting telephone interviews may apply stricter criteria for qualifying symptoms compared to participants self-reporting on a questionnaire. Additionally, our study focused on recent events within the past two months, whereas recall of symptoms from further back in time may be less reliable. Nonetheless, our findings as well as prior studies do suggest that stroke symptoms are relatively common in the general population. Although our survey data used to calculate the diagnostic value may be susceptible to information bias, prior clinical studies support the notion that dizziness/vertigo may be rather non-specific to stroke. For example, of patients attending the emergency department for dizziness, only 3% were deemed to have an acute cerebrovascular event [30]. The inherent difficulty for patients to distinguish vertigo from non-rotatory dizziness further underscores the challenges of incorporating these symptoms in public education on stroke [34].

Alternative strategies for improving public education for TIA and minor stroke could focus on symptom severity and transientness, rather than different types of symptoms. Despite improved knowledge on stroke symptoms following public education, better recognition of stroke symptoms does not necessarily lead to immediate help-seeking behavior when stroke symptoms arise [35]. The impact of public education efforts could be enhanced by highlighting the imperative of directly contacting medical services upon stroke symptom manifestation, even when symptoms are transient or occur in isolation. Seeking urgent medical assistance for transient cerebrovascular events is important, as early recurrence risk is as high as 10% within one week, and 50% of early recurrent strokes occur after a TIA for which no medical attention was sought [7]. Notably, the introduction of public education campaigns has not increased suspected cerebrovascular event presentations at general practitioners, indicating that public education fosters response directly to hospitals by emergency medical services rather than increasing suspected case numbers overall [14, 36, 37]. Half of patients with TIA present with isolated symptoms [7, 38]. These individuals delay longer in seeking medical attention than patients experiencing two or more symptoms [39, 40]. Indeed, those with isolated symptoms commonly refrain from seeking medical attention at all [7], despite similar short- and long-term risk of recurrent stroke compared to those with multiple symptoms [41]. Focus on urgency of response when transient or isolated symptoms occur may be crucial to improve the effectiveness of public education campaign for TIA and minor stroke.

Some limitations need to be taken into account when interpreting our findings. First, we did not interview patients in the acute phase after symptom onset. Imperfect recall of symptoms and incomplete annotation in medical records may have led to some under-recognition of symptoms. As these symptoms are most often FAST-negative [20], effects on FAST sensitivity are likely limited, but unheeded and unrecorded occurrence notably of inattention, ataxia, or sensory deficits may affect the choice and diagnostic value of FAST expansions. Second, although the demographic build-up of the Rotterdam Study population in terms of disease burden is similar to the Dutch population, extrapolation of incidence rates might prove an underestimation due to study screening for cardiometabolic risk factors or a Hawthorne effect, i.e., the change in behavior as a response to monitoring participants from our study [42]. In fact, the estimates number of strokes was only marginally lower than reported in nationwide statistics [43]. This may have led to a slight underestimation of the positive predictive values. Third, we used questionnaire data to estimate the incidence of transient neurological symptoms in the general population. Although surveys are the only feasible way to obtain these numbers, interpretation of the questions on focal neurological symptoms may have differed between participants, and bias these numbers in either direction.

In conclusion, the FAST-test has high sensitivity for major stroke, but fails to capture one-third of TIA and minor stroke events. Although sensitivity would increase substantially by incorporating visual symptoms and vertigo in the acronym, the large number of false positives could favor a focus on less severe and transient events, rather than additional symptoms, in future public education campaigns on stroke awareness.

## Supplementary Information

Supplementary Material 1.

## Data Availability

Data can be obtained upon request. Requests should be directed towards the management team of the Rotterdam Study (secretariat.epi@erasmusmc.nl), which has a protocol for approving data requests. Because of restrictions based on privacy regulations and informed consent of the participants, data cannot be made freely available in a public repository. FJW had full access to the data in the study and takes responsibility for data integrity and accuracy of data analysis.
