# Supplementary material for: Characterizing TIA and stroke symptomatology in a population-based study: implications for and diagnostic value of FAST-based public education
Source: BMC Public Health. 2024 Dec 18;24:3512. doi: 10.1186/s12889-024-20960-5 (PMC11657979; doi:10.1186/s12889-024-20960-5)
Supplement: Supplementary file 1 — Supplementary Material 1. [file 12889_2024_20960_MOESM1_ESM.docx]

**Supplemental material**

**Supplemental Figure 1.**

**Title: Flowchart of Patients Selected for Analysis**

All first TIA or stroke from 1 April 2002 – 1 January 2016

TIA 473

Minor stroke 292

Major stroke 368

Prevalent dementia: n=144

All first TIA or stroke, free of dementia

TIA 423

Minor stroke 263

Major stroke 303

Event with confusion or decreased consciousness: n=91

Population for analysis

TIA 409

Minor stroke 254

Major stroke 237

TIA indicates transient ischemic attack

**Supplemental Table 1.**

**Title: Detailed information on questionnaire neurological symptoms and help-seeking behavior**

The following questions are about the past 2 months. During the Covid-pandemic you might have had complaints or health issues. We would like to know how you responded to those complaints. These can be familiar complaints that gave rise to new problems, but could also be newly developed complaints.

|  | Did not have | Did have, and seeked contact with a physician | Did have, but did not seek contact with a physician due to COVID | Did have, but did not seek contact with a physician due to another reason | The other reason was … |
| --- | --- | --- | --- | --- | --- |
| Sudden vertigo |  |  |  |  |  |
| Loss of strength in arm or leg |  |  |  |  |  |
| Trouble speaking or facial droop |  |  |  |  |  |
| Sudden (short) loss of vision |  |  |  |  |  |

To what extent have you suffered from the following symptoms in the past two months?

Not at all A little Somewhat A fair amount Very much

A numb or tingling sensation anywhere in your body:*

*Sensory symptoms were classified as present when “somewhat”, “a fair amount” or “very much” was answered.
